# Supplementary material for: Punctuated mutagenesis promotes multi-step evolutionary adaptation in human cancers
Source: eLife. 2026 Oct 9;14:RP108058. doi: 10.7554/eLife.108058 (PMC13652540; doi:10.7554/eLife.108058)
Supplement: Peer review history

# Editor report: eLife Assessment

**DOI:** 10.7554/eLife.108058.3.sa0

**Authors:** Anna Panchenko

**Affiliations:** Queen's University Canada

**Keywords:** Solid, Valuable

This **valuable** study presents a theoretical model of how punctuated mutations influence multistep adaptation, supported by empirical evidence from some TCGA cancer cohorts. This **solid** model points to the case of possible punctuated evolution rather than gradual genomic change. There was some disagreement amongst the reviewers in terms of how closely the theoretical results apply to the phenomena examined empirically, and alternative explanations should be considered in the future.

# Referee report: Reviewer #1 (Public review)

**DOI:** 10.7554/eLife.108058.3.sa1

Summary:

Grasper et al. present a combined analysis of the role of temporal mutagenesis in cancer, which includes both theoretical investigation and empirical analysis of point mutations in TCGA cancer patient cohorts. They find that temporal elevated mutation rates contribute to cancer fitness by allowing fast adaptation when the fitness drops (due to previous deleterious mutations). This may be relevant in the case of tumor suppressor genes (TSG), which follow the 2-hit hypothesis (i.e., biallelic 2 mutations are necessary to deactivate TS), and in cases where temporal mutagenesis occurs (e.g. high APOBEC, ROS). They provide evidence that this scenario is likely to occur in patients, in some cancer types. This is an interesting and potentially important result that merits the attention of the target audience. Nonetheless, I have some questions (detailed below) regarding the design of the study, the tools and parametrization of the theoretical analysis and the empirical analysis - that I think if addressed would make the paper more solid and the conclusion more substantiated.

Strengths:

Combined theoretical investigation with empirical analysis of cancer patients

Weaknesses:

Parametrization and systematic investigation of theoretical tools and their relevant to tumor evolution

Comments on revisions:

The authors have adequately addressed my suggestions. I think some of the details provided in some of the replies to my comments (specifically with regard to my points 1, 4, 6ii; minor point 6) could be integrated into relevant text in the introduction , discussion and methods, to help the readers follow better the model and its interpretation - but this is up to the authors to decide what to emphasize.

# Referee report: Reviewer #2 (Public review)

**DOI:** 10.7554/eLife.108058.3.sa2

This work presents theoretical results concerning the effect of punctuated mutation on multistep adaptation along with empirical analysis of multistep adaptation in cancer. The empirical results are claimed to demonstrate the acceleration of multistep adaptation predicted theoretically. However, there is an important disconnect between the theoretical results and the empirical observations, such that it is not clear that punctuated mutation can produce the phenomena observed empirically. Furthermore, there are other plausible explanations for the empirical observations.

The theoretical work emphasizes the positive effect of punctuated mutation on the rate of crossing a "fitness valley", i.e., multistep adaptation where the first mutation is deleterious. The empirical work, however, focuses on inactivation of both alleles of a tumor suppressor gene (TSG), for which the first mutation--inactivation of one gene copy--is expected to be neutral or slightly advantageous, not maladaptive as suggested by the authors. Pairs of genes with putative synergystic effects were also analyzed, but there is no indication that these generally involve fitness valleys either.

This disconnect is most glaring in Figure 4, in which the simulations are supposed to confirm that punctuated mutation can produce the empirical phenomena reported for TSG inactivation. If this is the case, it should be possible to produce such results in simulations in which inactivation of just one allele is neutral. Instead, simulations assuming a substantial fitness penalty (0.05) for the first mutation are presented. Contrary to what is claimed in the text (line 212), this is not a "biologically realistic" parameter value for TSG inactivation. The insensitivity of results to the size the fitness penalty is irrelevant: a substantial fitness penalty is qualitatively different from no penalty at all.

The paper does report a small (15%) effect of punctuation on the rate of multistep adaptation in the absence of a fitness valley. This effect is much smaller than the fourfold increase in the presence of a fitness valley. The results presented--a single stochastic run for each condition--are insufficient to establish that there is any effect at all: if we assume that the number of pairs of fixations (about 150-180 in each simulation) is Poisson distributed, the 15% difference is not statistically significant.

Assuming that this effect is genuine, it is likely due to a mutation rate that is unrealisitcally high (considering that "rescue" requires inactivation of a particular gene). Theoretical considerations suggest that punctuated mutation has little or no effect in the absence of a fitness valley in the limit of low mutation rate:

(A1) The authors' theoretical results for a Galton-Watson process (SI2) imply that there is no effect without a fitness valley in that limit. This is so because there is no effect in the "supercritical" regime. Cancer cells must be supercritical (otherwise there would be no net growth), and a neutral or advantangeous mutant would remain in the supercritical regime.

(A2) Fig. S2D indicates, as far as I can tell from the colors, that punctuation makes little or no difference to the rate of adaptation in the absence of a fitness valley, i.e., for vertical axis values of 1 or more. I am not sure why the authors (line 129) point to this figure as evidence that punctuation speeds two-step adaptation when the first mutation is not maladaptive; the figure appears to say that it does not. The fraction of events due to "stochastic tunneling" of course increases with punctuation, but that does not change the fact that adaptation is no faster.

(A3) The authors' verbal argument to the contrary (line 124ff) is flawed. Despite the fact that even a mildly advantageous mutant is likely to go extinct, its expected frequency only increases with time, and that of a neutral allele remains constant over time. Thus, the average number of opportunities for a second mutation does not decrease with time since the first mutation, as it does when the first muation is deleterious.

(A4) I ran some simulations for a Wright-Fisher population, and they seem to confirm the lack of an effect in the low mutation rate limit.

Thus, it is unclear whether punctuated mutation can explain the reported phenomena or should be expected to have major effects on the rate or nature of cancer cell adaptation.

I would also note that routes to inactivation of both copies of a TSG that are not accelerated by punctuation will dilute any effects of punctuation. An example is a single somatic mutation followed by loss of heterozygosity. Such mechanisms are not included in the theoretical analysis nor assessed empirically. If, for example, 90% of double inactivations were the result of such mechanisms with a constant mutation rate, a factor of two effect of punctuated mutagenesis would increase the overall rate by only 10%. Consideration of the rate of apparent inactivation of just one TSG copy and of deletion of both copies would shed some light on the importance of this consideration.

Several factors besides the effects of punctuated mutation might explain or contribute to the empirical observations. Though these are now mentioned in the paper, I will list them here for clarity:

(B1) High APOBEC3 activity can select for inactivation of TSGs (references in Butler and Banday 2023, PMID 36978147). This could explain the empirical correlations.

(B2) Without punctuation, the rate of multistep adaptation is expected to rise more than linearly with mutation rate. Thus, if APOBEC signatures are correlated with a high mutation rate due to the action of APOBEC, this alone could explain the correlation with TSG inactivation.

(B3) The nature of mutations caused by APOBEC might explain the results. Notably, one of the two APOBEC mutation signatures, SBS13, is particularly likely to produce nonsense mutations. The authors count both nonsense and missense mutations, but nonsense mutations are more likely to inactivate the gene, and hence to be selected.

# Author comment: Author response

**DOI:** 10.7554/eLife.108058.3.sa3

**Authors:** Christopher J Graser, Wenbo Wu, Cole Christini, Mia Peljak, Franziska Michor

**Affiliations:** Dana-Farber Cancer Institute Boston United States; Dana-Farber Cancer Institute Boston United States; Dana-Farber Cancer Institute Boston United States; New York University School of Medicine New York United States; Dana-Farber Cancer Institute Boston United States

The following is the authors’ response to the original reviews

**eLife Assessment**

his valuable study presents a theoretical model of how punctuated mutations influence multistep adaptation, supported by empirical evidence from some TCGA cancer cohorts. This solid model is noteworthy for cancer researchers as it points to the case for possible punctuated evolution rather than gradual genomic change. However, the parametrization and systematic evaluation of the theoretical framework in the context of tumor evolution remain incomplete, and alternative explanations for the empirical observations are still plausible.

We thank the editor and the reviewers for their thorough engagement with our work. The reviewers’ comments have drawn our attention to several important points that we have addressed in the updated version. We believe that these modifications have substantially improved our paper.

There were two major themes in the reviewers’ suggestions for improvement. The first was that we should demonstrate more concretely how the results in the theoretical/stylized modelling parts of our paper quantitatively relate to dynamics in cancer.

To this end, we have now included a comprehensive quantification of the effect sizes of our results across large and biologically-relevant parameter ranges. Specifically, following reviewer 1’s suggestion to give more prominence to the branching process, we have added two figures (Fig S3-S4) quantifying the likelihood of multi-step adaptation in a branching process for a large range of mutation rates and birth-death ratios. Formulating our results in terms of birth-death ratios also allowed us to provide better intuition regarding how our results manifest in models with constant population size vs models of growing populations. In particular, the added figure (Fig S3) highlights that the effect size of temporal clustering on the probability of successful 2-step adaptation is very sensitive to the probability that the lineage of the first mutant would go extinct if it did not acquire a second mutation. As a result, the phenomenon we describe is biologically likely to be most effective in those phases during tumor evolution in which tumor growth is constrained. This important pattern had not been described sufficiently clearly in the initial version of our manuscript, and we thank both reviewers for their suggestions to make these improvements.

The second major theme in the reviewers’ suggestions was focused on how we relate our theoretical findings to readouts in genomic data, with both reviewers pointing to potential alternative explanations for the empirical patterns we describe.

We have now extended our empirical analyses following some of the reviewers’ suggestions. Specifically, we have included analyses investigating how the contribution of reactive oxygen species (ROS)-related mutation signatures correlates with our proxies for multi-step adaptation; and we have included robustness checks in which we use Spearman instead of Pearson correlations. Moreover, we have included more discussion on potential confounds and the assumptions going into our empirical analyses as well as the challenges in empirically identifying the phenomena we describe.

Below, we respond in detail to the individual comments made by each reviewer.

**Public Reviews:**

**Reviewer #1 (Public review):**

Summary:

Grasper et al. present a combined analysis of the role of temporal mutagenesis in cancer, which includes both theoretical investigation and empirical analysis of point mutations in TCGA cancer patient cohorts. They find that temporally elevated mutation rates contribute to cancer fitness by allowing fast adaptation when the fitness drops (due to previous deleterious mutations). This may be relevant in the case of tumor suppressor genes (TSG), which follow the 2-hit hypothesis (i.e., biallelic 2 mutations are necessary to deactivate TS), and in cases where temporal mutagenesis occurs (e.g., high APOBEC, ROS). They provide evidence that this scenario is likely to occur in patients with some cancer types. This is an interesting and potentially important result that merits the attention of the target audience. Nonetheless, I have some questions (detailed below) regarding the design of the study, the tools and parametrization of the theoretical analysis, and the empirical analysis, which I think, if addressed, would make the paper more solid and the conclusion more substantiated.

Strengths:

Combined theoretical investigation with empirical analysis of cancer patients.

Weaknesses:

Parametrization and systematic investigation of theoretical tools and their relevance to tumor evolution.

We sincerely thank Reviewer 1 for their comments. As communicated in more detail in the point-by-point replies to the “Recommendations for the authors”, we have revised the paper to address these comments in various ways. To summarize, Reviewer 1 asked for (1) more comprehensive analyses of the parameter space, especially in ranges of small fitness effects and low mutation rates; (2) additional clarifications on details of mechanisms described in the manuscript; and (3) suggested further robustness checks to our empirical analyses. We have addressed these points as follows: we have added detailed analyses of dynamics and effect sizes for branching processes (see Sections SI2 and SI3 in the Supplementary Information, as well as Figures S3 and S4). As suggested, these additions provide characterizations of effect sizes in biologically relevant parameter ranges (low mutation rates and smaller fitness effect sizes), and extend our descriptions to processes with dynamically changing population sizes. Moreover, we have added further clarifications at suggested points in the manuscript, e.g. to elaborate on the non-monotonicities in Fig 3. Lastly, we have undertaken robustness checks using Spearman rather than Pearson correlation coefficients to quantify relations between TSG deactivation and APOBEC signature contribution, and have performed analyses investigating dynamics of reactive oxygen species-associated mutagenesis instead of APOBEC.

**Reviewer #2 (Public review):**

This work presents theoretical results concerning the effect of punctuated mutation on multistep adaptation and empirical evidence for that effect in cancer. The empirical results seem to agree with the theoretical predictions. However, it is not clear how strong the effect should be on theoretical grounds, and there are other plausible explanations for the empirical observations.

Thank you very much for these comments. We have now substantially expanded our investigations of the parameter space as outlined in the response to the “eLife Assessment” above and in the detailed comments below (A(1)-A(3)) to convey more quantitative intuition for the magnitude of the effects we describe for different phases of tumor evolution. We agree that there could be potential additional confounders to our empirical investigations besides the challenges regarding quantification that we already described in our initial version of the manuscript. We have thus included further discussion of these in our manuscript (see replies to B(1)-B(3)), and we have expanded our empirical analyses as outlined in the response to the “eLife Assessment”.

For various reasons, the effect of punctuated mutation may be weaker than suggested by the theoretical and empirical analyses:

(A1) The effect of punctuated mutation is much stronger when the first mutation of a two-step adaptation is deleterious (Figure 2). For double inactivation of a TSG, the first mutation--inactivation of one copy--would be expected to be neutral or slightly advantageous. The simulations depicted in Figure 4, which are supposed to demonstrate the expected effect for TSGs, assume that the first mutation is quite deleterious. This assumption seems inappropriate for TSGs, and perhaps the other synergistic pairs considered, and exaggerates the expected effects.

Thank you for highlighting this discrepancy between Figure 2 and Figure 4. For computational efficiency and for illustration purposes, we had opted for high mutation rates and large fitness effects in Figure 2; however, our results are valid even in the setting of lower mutation rates and fitness effects. To improve the connection to Figure 4, and to address other related comments regarding parameter dependencies, we have now added more detailed quantification of the effects we describe (Figures SF3 and SF4) to the revised manuscript. These additions show that the effects illustrated in Figure 2 retain large effect sizes when going to much lower mutation rates and much smaller fitness effects. Indeed, while under high mutation rates we only see the large relative effects if the first mutation is highly deleterious, these large effects become more universal when going to low mutation rates.

In general, it is correct that the selective disadvantage (or advantage) conveyed by the first mutation affects the likelihood of successful 2-step adaptations. It is also correct that the magnitude of the ‘relative effect’ $R_{e}=a R_{f}+(1-a) R_{s}$ of temporal clustering on valley-crossing is highest if the lineage with only the first of the two mutations is vanishingly unlikely to produce a second mutant before going extinct. If the first mutation is strongly deleterious, the lineage of such a first mutant is likely to quickly go extinct – and therefore also more likely to do so before producing a second mutant.

However, this likelihood of producing the second mutant is also low if the mutation rate is low. As our added figure (Figure SF3) illustrates, at low mutation rates appropriate for cancer cells, $R_{e}=a R_{f}+(1-a) R_{s}$ is insensitive to the magnitude of the fitness disadvantage for large parts of the parameter space. Especially in populations of constant size (approximated by a birth/death ratio of 1), the relative effects $R_{e}=a R_{f}+(1-a) R_{s}$ for first mutations that reduce the birth rate by 0.5 or by 0.05 are indistinguishable (Figure SF3f).

Moreover, the absolute effect $\left(f_{k}-f_{1}\right)$, as we discuss in the paper (Figures SF2 and SF3) is largest in regions of the parameter space in which the first mutant is not infinitesimally unlikely to produce a second mutant (and 𝑓_𝑘_ and 𝑓_1_ would be infinitesimally small), but rather in parameter regions in which this first mutant has a non-negligible chance to produce a second mutant. The absolute effect $\left(f_{k}-f_{1}\right)$ therefore peaks around fitness-neutral first mutations. While the next comment (below) says that our empirical investigations more closely resemble comparisons of relative effects and not absolute effects, we would expect that the observations in our data come preferentially from multi-step adaptations with large absolute effect since the absolute effect is maximal when both 𝑓_𝑘_ and *f*_1_are relatively high.

In summary, we believe Figure 2, while having exaggerated parameters for very defendable reasons, is not a misleading illustration of the general phenomenon or of its applicability in biological settings, as effect sizes remain large when moving to biologically realistic parameter ranges. To clarify this issue, we have largely rewritten the relevant paragraphs in the results section and have added two additional figures (Figures SF3 and SF4) as well as a section in the SI with detailed discussion (SI2).

(A2) More generally, parameter values affect the magnitude of the effect. The authors note, for example, that the relative effect decreases with mutation rate. They suggest that the absolute effect, which increases, is more important, but the relative effect seems more relevant and is what is assessed empirically.

Thank you for this comment. As noted in the replies to the above comments, we have now included extensive investigations of how sensitive effect sizes are to different parameter choices. We also apologize for insufficiently clearly communicating how the quantities in Figure 4 relate to the findings of our theoretical models.

The challenge in relating our results to single-timepoint sequencing data is that we only observe the mutations that a tumor has acquired, but we do not directly observe the mutation rate histories that brought about these mutations. As an alternative readout, we therefore consider (through rough proxies: TSGs and APOBEC signatures) the amount of 2-step adaptations per acquired/retained mutation. While we unfortunately cannot control for the average mutation rate in a sample, we motivate using this “TSG-deactivation score” by the hypothesis that for any given mutation rate, we expect a positive relationship between the amount of temporal clustering and the amount of 2-step adaptations per acquired/retained mutation. This hypothesis follows directly from our theoretical model where it formally translates to the statement that for a fixed , is increasing in .

However, while both quantities 𝑓_𝑘_/𝑓_1_ or $\left(f_{k}-f_{1}\right)$ from our theoretical model relate to this hypothesis – both are increasing in 𝑘–, neither of them maps directly onto the formulation of our empirical hypothesis.

We have now rewritten the relevant passages of the manuscript to more clearly convey our motivation for constructing our TSG deactivation score in this form (P. 4-6).

(A3) Routes to inactivation of both copies of a TSG that are not accelerated by punctuation will dilute any effects of punctuation. An example is a single somatic mutation followed by loss of heterozygosity. Such mechanisms are not included in the theoretical analysis nor assessed empirically. If, for example, 90% of double inactivations were the result of such mechanisms with a constant mutation rate, a factor of two effect of punctuated mutagenesis would increase the overall rate by only 10%. Consideration of the rate of apparent inactivation of just one TSG copy and of deletion of both copies would shed some light on the importance of this consideration.

This is a very good point, thank you. In our empirical analyses, the main motivation was to investigate whether we would observe patterns that are qualitatively consistent with our theoretical predictions, i.e. whether we would find positive associations between valley-crossing and temporal clustering. Our aim in the empirical analyses was not to provide a quantitative estimate of how strongly temporally clustered mutation processes affect mutation accumulation in human cancers. We hence restricted attention to only one mutation process which is well characterized to be temporally clustered (APOBEC mutagenesis) and to only one category of (epi)genomic changes (SNPs, in which APOBEC signatures are well characterized). Of course, such an analysis ignores that other mutation processes (e.g. LOH, copy number changes, methylation in promoter regions, etc.) may interact with the mechanisms that we consider in deactivating Tumor suppressor genes.

We have now updated the text to include further discussion of this limitation and further elaboration to convey that our empirical analyses are not intended as a complete quantification of the effect of temporal clustering on mutagenesis in-vivo (P. 10,11).

Several factors besides the effects of punctuated mutation might explain or contribute to the empirical observations:

(B1) High APOBEC3 activity can select for inactivation of TSGs (references in Butler and Banday 2023, PMID 36978147). This selective force is another plausible explanation for the empirical observations.

Thank you for making this point. We agree that increased APOBEC3 activity, or any other similar perturbation, can change the fitness effect that any further changes/perturbations to the cell would bring about. Our empirical analyses therefore rely on the assumption that there are no major confounding structural differences in selection pressures between tumors with different levels of APOBEC signature contributions. We have expanded our discussion section to elaborate on this potential limitation (P. 10-11).

While the hypothesis that APOBEC3 activity selects for inactivation of TSGSs has been suggested, there remain other explanations. Either way, the ways in which selective pressures have been suggested to change would not interfere relevantly with the effects we describe. The paper cited in the comment argues that “high APOBEC3 activity may generate a selective pressure favoring” TSG mutations as “APOBEC creates a high [mutation] burden, so cells with impaired DNA damage response (DDR) due to tumor suppressor mutations are more likely to avert apoptosis and continue proliferating”. To motivate this reasoning, in the same passage, the authors cite a high prevalence of TP53 mutations across several cancer types with “high burden of APOBEC3-induced mutations”, but also note that “this trend could arise from higher APOBEC3 expression in p53-mutated tumors since p53 may suppress APOBEC3B transcription via p21 and DREAM proteins”.

Translated to our theoretical framework, this reasoning builds on the idea that APOBEC3 activity increases the selective advantage of mutants with inactivation of both copies of a TSG. In contrast, the mechanism we describe acts by altering the chances of mutants with only one TSG allele inactivated to inactivate the second allele before going extinct. If homozygous inactivation of TSGs generally conveys relatively strong fitness advantages, lineages with homozygous inactivation would already be unlikely to go extinct. Further increasing the fitness advantage of such lineages would thus manifest mostly in a quicker spread of these lineages, rather than in changes in the chance that these lineages survive. In turn, such a change would have limited effect on the “rate” at which such 2-step adaptations occur, but would mostly affect the speed at which they fixate. It would be interesting to investigate these effects empirically by quantifying the speed of proliferation and chance of going extinct for lineages that newly acquired inactivating mutations in TSGs.

Beyond this explicit mention of selection pressures, the cited paper also discusses high occurrences of mutations in TSGs in relation to APOBEC. These enrichments, however, are not uniquely explained by an APOBEC-driven change in selection pressures. Indeed, our analyses would also predict such enrichments.

(B2) Without punctuation, the rate of multistep adaptation is expected to rise more than linearly with mutation rate. Thus, if APOBEC signatures are correlated with a high mutation rate due to the action of APOBEC, this alone could explain the correlation with TSG inactivation.

Thank you for making this point. Indeed, an identifying assumption that we make is that average mutation rates are balanced between samples with a higher vs lower APOBEC signature contribution. We cannot cleanly test this assumption, as we only observe aggregate mutation counts but not mutation rates. However, the fact that we observe an enrichment for APOBEC-associated mutations among the set of TSG-inactivating mutations (see Figure 4F) would be consistent with APOBEC-mutations driving the correlations in Fig 4D, rather than just average mutation rates. We have now added a paragraph to our manuscript to discuss these points (P. 10-11).

(B3) The nature of mutations caused by APOBEC might explain the results. Notably, one of the two APOBEC mutation signatures, SBS13, is particularly likely to produce nonsense mutations. The authors count both nonsense and missense mutations, but nonsense mutations are more likely to inactivate the gene, and hence to be selected.

Thank you for making this point. We have included it in our discussion of potential confounders/limitations in the revised manuscript (P. 10-11).

**Recommendations for the authors:**

**Reviewer #1 (Recommendations for the authors):**

Specific questions/comments/suggestions:

(1) For the theoretical investigation, the authors use the Wright-Fisher model with specific parameters for the decrease/increase in the fitness (0.5,1.5). This model is not so relevant to cancer, because it assumes a constant population size, while in cancer, the population is dynamic (increasing, if the tumor grows). Although I see they mention relevance to the branching process (in SI), I think the branching process should be bold in the main text and the Wright-Fisher in SI (or even dropped).

Thank you for this comment. We agree that too little attention had been given to the branching process in the original version of our manuscript. While the Wright-Fisher process is computationally efficient to simulate and thus lends itself to clean simulations for illustrative examples, it did lead us to put undue emphasis on populations of constant size.

The added Figures SF2 and SF3 now focus on branching processes, and we have substantially expanded our discussion of how dynamics differ as a function of the population-size trajectory (constant vs growing; SI2, P. 4,9,10). Generally, we do believe that it is appropriate to consider both regimes. If tumors evolve from being confined within their site of origin to progressively invading adjacent tissues and organ compartments, they traverse different regions of the birth-death ratio parameter space. Moreover, the timing of transitions between phases of more or less constrained growth is likely closely tied to adaptation dynamics, since breaching barriers to expansion requires adapting to novel environments and selection pressures.

We hope that the revised version of the manuscript conveys these points more clearly, and thank you for alerting us to this imbalance in the original version of our manuscript.

(2) The parameters 0.5 (decrease in fitness) and 1.5 (increase in fitness) seem exaggerated (the typical values for the selective advantage are usually much lower (by an order of magnitude)). The same goes for the mutation rate. The authors chose values of the order 0.001, while in cancer (and generally) it is much lower than that (10-5 - 10-6). I think that generally, the authors should present a more systematic analysis of the sensitivity of the results to these parameters.

Thank you very much for this very important comment. We have made this a major focus in our revisions (see our reply to the editor’s comments). As suggested, we have now added further analyses to explore more biologically relevant parameter regimes. Reviewer 2 has made a similar remark, and to avoid redundancies, we point for a more detailed response to our response to that comment (A1).

(3) In Figure 3, the authors explore the sensitivity to mu (mutation rate) and k (temporal clustering) and find a non-monotonic behavior (Figure 3C). However, this behavior is not well explained. I think some more explanations are required here.

Thank you for pointing this out. We had initially relegated the more detailed explanations to the SI2 (which in the revised manuscript became SI4), but are happy to provide more elaboration in the main text, and have done so now (P. 5).

For , the non-monotonicity reflects the exploration-exploitation tradeoff that this section is dedicated to very small values (little exploration) prevent the population from finding fitness peaks. In contrast, once a fitness peak is reached, excessively large values (little exploitation) scatter the population away from this peak to points of lower fitness.

For , the most relevant dynamic is that at high , the population becomes unable to find close-by fitness improvements (1-step adaptations) if it is not in a burst. As 𝑘 increases, this delay in adaptation (until a burst occurs) eventually comes to outweigh the benefits of high 𝑘 (better ability to undergo multi-step adaptations). Additionally, if 𝑘 ∙ μ becomes very large, clonal interference eventually leads to diminishing exploration-returns when 𝑘 is increased further (Fig 5C), as the per-cell likelihood of finding a specific fitness peak eventually saturates and increasing only causes multiple cells to find the same peak, rather than one cell finding this peak and its lineage fixating in the population.

(4) In Figure 5, where the authors show the accumulation of the first (red; deleterious mutation) and second (blue; advantageous mutation), it seems that the fraction of deleterious mutations is much lower than that of advantageous mutations. This is opposite to the case of cancer, where most of the mutations are 'passengers', (slightly) deleterious or neutral mutations. Can the author explain this discrepancy and generally the relation of their parametrization to deleterious vs. advantageous mutations?

Thank you for this comment. In general, we have focused attention in our paper on sequences of mutations that bring about a fitness increase. We call those sequences ‘adaptations’ and categorize these as one-step or multi-step, depending on whether or not they contain intermediates states with a fitness disadvantage.

In our modelling, we do not consider mutations that are simply deleterious and are not a necessary part of a multi-step adaptation sequence. The motivation for this abstraction is, firstly, to focus on adaptation dynamics, and secondly, that in certain limits (small mu and large constant population sizes), lineages with only deleterious mutations have a probability close to one of going extinct, so that any emerging deleterious mutant would likely be 'washed out’ of the population before a new mutation emerges.

However, whether the dynamics of how neutral or deleterious passenger mutations are acquired also vary relevantly with the extent of temporal clustering is a valid and interesting question that would warrant its own study. The types of theoretical arguments for such an investigation would be very similar to the ones we use in our paper.

(5) The theoretical investigation assumes a multi/2-step adaptation scenario where the first mutation is deleterious and the second is advantageous. I think this should be generalized and further explored. For example, what happens when there are multiple mutations that are slightly deleterious (as probably is the case in cancer) and only much later mutations confer a selective advantage? How stable is the "valley crossing" if more deleterious mutations occur after the 2 steps?

This is also an important point and relates in part to the previous comment (4). For discussion of interactions with deleterious mutations, please see the reply to comment (4).

Regarding generalizations of this valley-crossing scenario, note that any sequence of mutations that increases fitness can be decomposed into sequences of either one-step or multi-step adaptations, as defined in the paper. Therefore, if all intermediate states before the final selectively advantageous state have a selective disadvantage making the lineages of such cells likely to go extinct, then our derivations in S1 apply, and the relative effect of temporal clustering becomes $\frac{f_{k}}{f_{1}}=k^{n}$ where n is the number of intermediate states. If, conversely, any of the intermediate states already had a selective advantage, then our model would consider the subsequence until this first mutation with a selective advantage as its individual (one-step or multi-step) “adaptation”.

The second question, “How stable is the "valley crossing" if more deleterious mutations occur after the 2 steps?”, touches on a different property of the population dynamics, namely on how the fate of a mutant lineage depends on how this lineage emerged. In our paper, we compare different levels of temporal clustering for a *fixed* average mutation rate. This choice implies that, if we assume that the mutant that emerges from a valley-crossing does not go extinct, then the number of deleterious mutations expected to occur in this lineage, once emerged, will not depend on the extent of temporal clustering. However, if in-burst mutation rates increased the expected burden of early acquired deleterious mutations sufficiently much to affect the probability that the lineage with a multi-step adaptation goes extinct before the burst ends, then there may indeed be an interaction between effects of deleterious passengers and temporal clustering. We would, however, expect effects on this probability of early extinction to be relatively minor, since such a lineage with a selective advantage would quickly grow to large cell-numbers implying that it would require a large number of co-occurring and sufficiently deleterious mutations across these cells for the lineage to go extinct.

(6) For the empirical analysis of TCGA cohorts, the authors focus on the contribution of APOBEC mutations (via signature analysis) to temporal mutagenesis. They find only a few cancer types (Figure 4D) that follow their prediction (in Figure 4C) of a correlation between TSG deactivation and temporal mutations in bursts. I think two main points should be addressed:

Thank you for this comment. We will respond in detail to the corresponding points below, but would like to note here that while we find this correlation “in only a few cancer types”, we also show that only few cancer types have relevant proportions of mutations caused by APOBEC, and it is precisely in these cancer types that we find a correlation. We have clarified this aspect in the revised version of the manuscript (P.7).

(i) APOBEC is not the only cause for temporal mutagenesis. For example, elevated ROS and hypoxia are also potential contributors - it might therefore be important to extend the signature analysis (to include more possible sources for temporal mutagenesis). Potentially, such an extension may show that more cancer types follow the author's prediction.

Thank you for this interesting suggestion. We have now included analogous analyses for contributions of signature SBS18 which is associated with ROS mutagenesis, and for the joint contribution of signatures SBS17a, SBS17b, SBS18 and SBS36, which all have been shown (some in a more context-dependent manner) to be associated with ROS mutagenesis. When doing so, we do not find a clear trend. However, we also do not find these signatures to account for substantial proportions of the acquired mutations, meaning that ROS mutagenesis likely also does not account for much of the variation in how temporally clustered the mutation rate trajectories of different tumors are. We have incorporated these results and their discussion in the manuscript (SI5 and Fig S8).

(ii) The TSG deactivation score used by the authors only counts the number of mutations and does not consider if the 2 mutations are biallelic, which is highly important in this case. There are ways to investigate the specific allele of mutations in TCGA data (for example, see Ciani et al. Cell Sys 2022 PMID: 34731645). Given the focus on TSG of this study, I think it is important to account for this in the analysis.

Thank you for making this point. We did initially consider inferring allele-specific mutation status, but decided against it as this would have shrunk our dataset substantially, thus potentially introducing unwanted biases. Determining whether two mutations lie on the same or on different alleles requires either (1) observing sequencing reads that either cover the loci of both mutations, or (2) tracing whether (sets of) other SNPs on the same gene co-occur exclusively with one of the two considered mutations. These requirements lead to a substantial filtering of the observed mutations. Moreover, this filtering would be especially strong for tumors with a small overall mutation burden, as these would have fewer co-occurring SNPs to leverage in this inference. We would have hence preferentially filtered out TSG-deactivating mutations in tumors with low mutation burden. We have modified the text to address this point (P.14).

(7) To continue point 4. I wonder why some known cancer types with high APOBEC signatures (e.g., lung, mentioned in the introduction) do not appear in the results of Figure 4. Can the author explain why it is missed?

We do provide complete results for all categories in Supplementary Figure 3. To not overwhelm the figure in the main text, we only show the four categories with the highest average APOBEC signature contribution, beyond those four, average APOBEC signature contributions quickly drop. Lung-related categories do not feature in these top four (Lung squamous cell carcinoma are fifth and Lung adenocarcinoma are eighth in this ordering).

Minors:

(1) It is worth mentioning the relevance to resistance to treatment (see https://www.nature.com/articles/s41588-025-02187-1).

Thank you for this suggestion. We have included a mention of the relation to this paper in the discussion section (P. 11).

(2) Some of the figures' resolution should be improved - specifically, Figures 4, S1, and S5, which are not clear/readable.

Thank you for pointing this out. This was the result of conversion to a word document. We will provide tif files in the revisions to have better resolution.

(3) Regarding Figure 3e,f. How come that moving from K=1 to K=I doesn't show any changes in fitness - it looks as if in both cases the value fluctuates around comparable mean fitness? Is that the case?

While fitness differences between simulations with different k manifest robustly over long time-horizons (see Fig 3C with results over generations), there are various sources of substantial stochasticity that make the fitness values in these short-term plots (Fig3D-F) imperfect illustrations of how long-term average fitness behaves. For instance, fitness landscapes are drawn randomly which introduces variability in how high and how close-by different fitness peaks are. Similarly, there is substantial randomness since both the type (direction on the 2-D fitness landscape) and the timing of mutation are stochastic.

The short-term plots in Fig3D-F are intended to showcase representative dynamics of transitions between points on the genotype space with different fitness values following a redrawing of the landscape – but not necessarily to provide a comparison between the height of the attained (local) fitness-maxima.

(4) Figures 4c,d - correlation should be Spearman, not Pearson (it's not a linear relationship).

Thank you for this comment. As a robustness check, we have generated the same figures using Spearman and not Pearson correlations and find results that are qualitatively consistent with the initially shown results. Indeed, using Spearman correlations, all four cancer types from Fig 4D have significant correlations.

(5) Typo for E "...in samples of the cancer types in (C) were caused by APOBEC" - it should be D (not C) I guess.

Thank you for catching this. We fixed the typo.

(6) Figure 5 - the mutation rate is too high (0.001), sensitivity to that? Also the fitness change is exaggerated (0.5, 1.5), and the division of mutations to 100 and 100 (200 in total) loci is not clear.

Thank you for making this point. In this simulation setting it is unfortunately computationally prohibitively expensive to perform simulations at biologically realistic mutation rates. Therefore, we have scaled up the mutation rate while scaling down the population size. Moreover, the choice of model here is not meant to resemble a biologically realistic dynamic, but rather to create a stylized setting to be able to consider the interplay between clonal interference and facilitated valley-crossing in isolation. The key result from this figure is the separation of time scales at which low or high temporal clustering maximizes adaptability.

However, known parameter dependencies in these models allow us to reason about how tuning individual parameters of this stylized model would affect the relative importance of effects of clonal interference. This relative importance is largest when mutants are likely to co-occur on different competing clones in a population. The likelihood of such co-occurrences decreases substantially if decreasing the mutation rate to biologically realistic values. However, this likelihood also sensitively depends on the time that it takes a clone with a one-step adaptation to spread through the population. Smaller fitness advantages, as well as larger population sizes, slow down this process of taking over the population, which increases the likelihood of clonal interference. We now discuss these points in our revised manuscript (P. 8).

7) In the results text (last section) "Performing simulations for 2-step adaptations, we found that fixation rates are non-monotone in k. While at low k increasing k leads to a steep increase in the fixation rate, this trend eventually levels off and becomes negative, with further increases in k leading to a decrease in the fixation rate". Where are the results of this? It should be bold and apparent.

Thank you for alerting us that this is unclear. The relevant figure reference is indeed Fig 5C as in the preceding passage in the manuscript. However, we noticed that due to the presence of the steadily decreasing black line for 1-step adaptations, it is not easy to see that also the blue line is downward sloping. We have added a further reference to Fig 5C, and have adapted the grid spacing in the background of that figure-panel to make this trend more easily visible.

(8) Although not inconceivable, conclusions regarding resistance in the discussion are overstated. If you want to make this statement, you need to show that in resistant tumors, the temporal mutagenesis is responsible for progression vs. non-resistant/sensitive cases (is that the case), otherwise this should be toned down.

Thank you for pointing this out. We have tempered these conclusions in the revised version of the manuscript (P. 11).

**Reviewer #2 (Recommendations for the authors):**

(1) It might be useful to look specifically at X-linked TSGs. On the authors' interpretation, their relative inactivation rates should not be correlated with APOBEC signatures in males (but should be in females), though the size of the dataset may preclude any definite conclusions.

Thank you for this suggestion. Indeed, the size of the dataset unfortunately makes such analyses infeasible. Moreover, it is not clear whether X-linked TSGs might have structurally different fitness dynamics than TSGs on other chromosomes. However, this is an interesting suggestion worth following up on as more synergistic pairs confined to the X-chromosome are getting identified.

(2) Might there be value in distinguishing tumors that carry mutations expected to increase APOBEC expression from those that do not? Among several reasons, an APOBEC signature due to such a mutation and an APOBEC signature due to abortive viral infection may differ with respect to the degree of punctuation.

This is also an interesting suggestion for future investigations, but for which we unfortunately do not have sufficient information to build a meaningful analysis. In particular, it is unclear to what extent the degree and manifestation of episodicity/punctuation varies between these different mechanisms. Burst duration and intensity, as well as out-of-burst baseline rates of APOBEC mutagenesis likely differ in ways that are yet insufficiently characterized, which would make any result of analyses like these in Fig 4 hard to interpret.

(3) Also, in that paragraph, is "proportional to" used loosely to mean "an increasing function of"?

Thank you for this comment. We are not quite sure which paragraph is meant, but we use the term “proportional” in a literal sense at every point it is mentioned in the paper.

For the occurrences of the term on pages 3, 10 and 11, the word is used in reference to probabilities of reproduction (division in the branching process, or ‘being drawn to populate a spot in the next generation’ in the WF process) being “proportional” to fitness. These probabilities are constructed by dividing each individual cell’s fitness by the total fitness summed across all cells in the population. As the population acquires fitness-enhancing mutations, the resulting proportionality constant (1/total_fitness) changes, so that the mapping from ‘fitness’ to probability of reproduction in the next reproduction event changes over time. Nevertheless, this mapping always remains fitness-proportional.

On page 4, the term is used as follows: “the absolute rates 𝑓_𝑘_ and 𝑓_1_ are proportional to *µ^n^*+1”. Here, proportionality in the literal sense follows from the equations on page 20, when setting $\tilde{\mu}=N \mu$, so that the second factor becomes *µ^n^*+1. We have included a clarifying sentence to address this in the derivations (SI1).

(4) It could be mentioned in the main text that the time between bursts (d) must not be too short in order for the effect to be substantial. I would think that the relevant timescale depends on how deleterious the initial mutation is.

Thank you for making this interesting and very relevant point. We have included a section (SI3) and Figure (Fig S4**)** in the supplement to investigate the dependence on d. In short, we find that effects are weaker for small inter-burst intervals. The sensitivity to the burst size is highest for inter-burst intervals that are sufficiently small so that the lineage of the first mutant has relevant probability of surviving long enough to experience multiple burst phases.

(5) Why not report that relative rate for Figure 2E as for 2D, as the former would seem to be more relevant to TSGs? And why was it assumed that the first inactivation is deleterious in the simulations in Figure 4 if the goal is to model TSGs?

Thank you for noting this. For how we revised the paper to better connect Figures 2 and 4, please see our comment (A1) above. In general, neither 2E nor 2D should serve as quantitative predictions for what effect size we should expect in real world data, but are rather curated illustrations of the general phenomenon that we describe: we chose high mutation rates and exaggerated fitness effects so that dynamics become visually tractable in small simulation examples.

For figure 4, assuming that the first inactivation is deleterious achieves that the branching process for the mutant lineage becomes subcritical, which keeps the simulation example simple and illustrative. For more comprehensive motivation of the approach in 4D, and especially the discussion of how fitness effects of different magnitudes may or may not be subject to the effects we describe depending on whether the population is in a phase of constant or growing population size, we refer the reader to our added section SI2, and the added discussion on pages 6 and 10.

(6) Figure 2, D and E. I'm not sure why heatmaps with height one were provided rather than simple plots over time. It is difficult, for example, to determine from a heatmap whether the increase is linear or the relative rates with and without punctuation.

Thank you for this comment. These are not heatmaps with height one, but rather for every column of pixels, different segments of that column correspond to different clones within that population. This approach is intended to convey the difference in dynamics between the results in Fig 2 and the analogous results for a branching process in Fig S1. In Fig 2, valley-crossings happen sequentially, with subsequent fixations of adapted mutants. In Fig S1, with a growing population size, multiple clones with different numbers of adaptations coexist. We have now adapted the caption of Fig 2 to clarify this point.

(7) Page 3: "High mutation rates are known to limit the rate of 1-step adaptations due to clonal interference." This is a bit misleading, as it makes it sound like increasing the mutation rate decreases the rate of one-step adaptations.

Thank you for alerting us to this poor phrasing. We have changed it in the revised version of the manuscript (P. 3).

(8) Page 4: "proportional to \mu^{n+1}" Is "proportional" being used loosely for "an increasing function of"?

It is meant in the literal mathematical sense (see response to comment (3))

(9) Page 5, near bottom: "at least two mutations across the population". In the same genome?

We counted mutations irrespective of whether they emerged in the same genome, to remain analogous to the TCGA analyses for which we also do not have single cell-resolved information.

(10) Page 6: "missense or nonsense mutation". What about indels? If these are not affected by APOBEC, omitting them will exaggerate the effect of punctuation.

Thank you for pointing out that this focus on single nucleotide substitutions conveys an exaggerated image of the importance of this effect of APOBEC-driven mutagenesis. There are of course several other classes of (epi)genomic alterations (e.g. chromatin modifications, methylation changes, copy number changes) that we do not consider in this part of our analysis. APOBEC mutagenesis serves as an example of a temporally clustered mutation process, which we investigate in its domain of action.

We have added further discussion (P. 10-11) to convey that our empirical results merely constitute an investigation of whether empirical patterns are consistent with our hypothesis, but that the narrow focus on only SNVs, only TSGs, and only APOBEC mutagenesis does not allow for a general quantitative statement about the in-vivo relevance of the phenomena we describe.

(11) Page 6: "normalized by the total number of single nucleotide substitutions." It is difficult to know how to normalize correctly, but I might think that the square of the number of substitutions would be more appropriate. Perhaps the total numbers are close enough that it matters little.

Thank you for noting this. In the revised manuscript we have now expanded this passage in the text to more clearly convey our motivations for why we normalize by the total number of single nucleotide substitutions. While the likelihood for crossing a fitness valley with 2 mutations is indeed proportional to the square of the mutation rate, we do not directly observe mutation rates from our data. Rather, we observe the number of acquired single nucleotide substitutions for every tumor sample, but since tumors in our data differ in the time since initiation and therefore differ in the numbers of divisions their cells have undergone before being sequenced, we cannot directly infer mutation rates. One way to phrase our main result about valley-crossing is that temporally clustered mutation processes have an increased rate of successful valley-crossings per attempted valley crossing. Our TSG deactivation score is constructed to reflect this idea. The number of TSGs serves as a proxy for successful valley-crossings and the total mutation burden serves as a proxy for attempted valley-crossings.

To convey these points more clearly, we have rewritten the first paragraph in the Section “Proxies for valley crossing and for temporal clustering found in patient data” (P.6)

(12) Perhaps embed links to the COSMIC web pages for SBS2 and SBS13 in the text.

Thank you for this suggestion. We have embedded the links at the first mention of SBS2 and SBS13 in the text.
